# Optimization of Oyster (*Crassostrea talienwhanensis*) Protein Hydrolysates Using Response Surface Methodology

**DOI:** 10.3390/molecules25122844

**Published:** 2020-06-19

**Authors:** Xueqin Wang, Huahua Yu, Ronge Xing, Song Liu, Xiaolin Chen, Pengcheng Li

**Affiliations:** 1 Key Laboratory of Experimental Marine Biology, Center for Ocean Mega-Science, Institute of Oceanology, Chinese Academy of Sciences, Qingdao 266071, China; xueqinwang@qdio.ac.cn (X.W.); yuhuahua@qdio.ac.cn (H.Y.); xingronge@qdio.ac.cn (R.X.); sliu@qdio.ac.cn (S.L.); chenxl@qdio.ac.cn (X.C.); 2 Laboratory for Marine Drugs and Bioproducts, Pilot National Laboratory for Marine Science and Technology (Qingdao), No. 1 Wenhai Road, Qingdao 266237, China

**Keywords:** oyster, protein hydrolysate, response surface methodology, degree of hydrolysis, hydroxyl-radical-scavenging activity, branched-chain amino acids

## Abstract

Oyster (*Crassostrea talienwhanensis*) protein was hydrolyzed by trypsin to produce peptides with different response values, and response surface methodology (RSM) was applied to optimize the hydrolysis conditions. The highest degree of hydrolysis (DH) of the oyster peptide (OP) was obtained at an enzyme concentration of 1593.2 U/g, a pH of 8.2, a hydrolysis temperature of 40.1 °C, a hydrolysis time of 6.0 h, and a water/material ratio of 8.2. The greatest hydroxyl-radical-scavenging activity of OP was obtained at an enzyme concentration of 1546.3 U/g, a pH of 9.0, a hydrolysis temperature of 50.2 °C, a hydrolysis time of 5.1 h, and a water/material ratio of 5.6. The largest branched-chain amino acid (BCAA) content of OP was obtained at an enzyme concentration of 1323.8 U/g, a pH of 8.3, a hydrolysis temperature of 41.7 °C, a hydrolysis time of 6.7 h, and a water/material ratio of 4.8. The three experimental values were significantly in agreement with the predicted values within the 95% confidence interval. Furthermore, ultrafiltration and chromatographic methods were used to purify the OP, and 13 peptides that were rich in Lys, Arg, His, and Thr were identified by LC-MS/MS. The results of this study offer different optimum hydrolysis conditions to produce target peptides from oyster protein by using RSM, and this study provide a theoretical basis for the high-value utilization of oyster protein.

## 1. Introduction

Oysters, as one of the most widely distributed marine biological resources worldwide, are rich in nutrients, including special amino acids, taurine, and trace elements. In many parts of the world, fresh oyster consumption habits exist because their meat is rich and tender, and oysters are processed into a variety of products, such as dried oysters and oyster glycogen [1]. Additionally, oysters have been shown to have many biological activities, e.g., Zha et al. [2] reported that the Hong Kong oyster (*Crassostrea hongkongensis*) has high acetylcholinesterase (AChE) activity, Wang et al. [3] confirmed that oyster (*Crassostrea gigas*) hydrolysates had anti-tumor activity and immunostimulatory effects in BALB/c mice, Lee et al. [4] studied the anti-inflammatory effects of oyster shells, and Miao et al. [5] found that oyster (*Ostrea rivularis*) hydrolysates had anti-fatigue and antioxidant activities. In addition, our previous studies found that oyster (*Crassostrea talienwhanensis*) protein hydrolysates could improve the spatial learning and memory capacity [6]. Although oyster extracts contain many bioactive components, there has been no systematic evaluation of the extraction conditions for oyster protein hydrolysates according to different applications.

Some studies have reported that the functional and bioactive properties of peptides are directly linked to their structural features, including molecular size, presence or absence of charges, amino acid sequence, hydrophobicity, and hydrophilicity [7]. In addition, the functional properties of protein hydrolysates also depend on the degree of hydrolysis (DH), which affects the size and hence the amino-acid composition of the peptides [8]. Protein hydrolysates with different DHs have different solubilities and emulsifying characteristics, foaming properties, and taste characteristics, and also have different antioxidant capacities [8]. While a high DH may not coincide with a high amount of hydrolysates, uncontrolled or prolonged hydrolysis of proteins may result in a low content of active peptides [9]. Therefore, controlling the DH during the protease hydrolysis process is related to the yield and activity of the peptides. Moreover, different hydrolysis conditions allow peptides with different properties to be obtained [10]. Thus, it is important to choose the appropriate conditions during hydrolysis.

Response surface methodology (RSM), a useful technique for investigating complex processes, has been successfully applied to optimize processing conditions such as enzyme concentration, pH, and hydrolysis temperature [11]. In addition, this experimental methodology can be used to evaluate effective factors and generate a mathematical model [12]. The objective of this study was to investigate the effect of hydrolysis conditions (enzyme concentration, pH, hydrolysis temperature, hydrolysis time, water/material ratio) on the hydroxyl-radical-scavenging activity and contents of DH and branched-chain amino acids (BCAAs) of oyster protein hydrolysates using RSM.

## 2. Results

### 2.1. Selection of Proteolytic Enzymes

**Figure 1:**
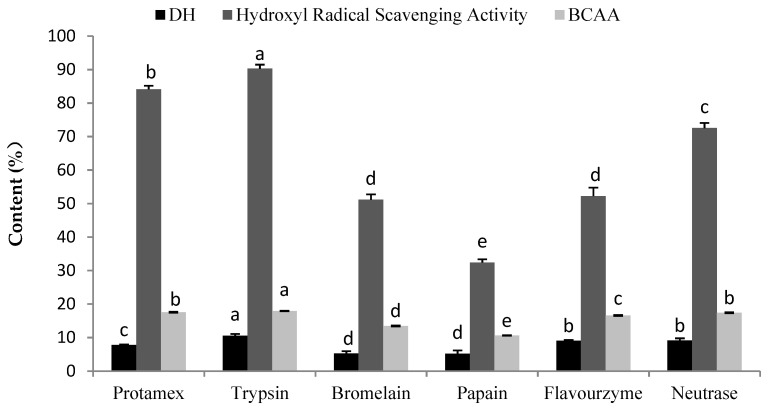
DH, hydroxyl-radical-scavenging activity, and BCAA contents of hydrolysates produced by various proteases. DH indicates the degree of hydrolysis; BCAA indicates branched-chain amino acids. The concentration of hydrolysates was 10 mg/mL in the hydroxyl-radical-scavenging activity assay. Different letters indicate significant differences between groups (*p* < 0.05).

It is important to point out that the addition of exogenous enzymes is the main contributor to the cost of a hydrolysis process [13]. The types of proteases used affect the DH and play an important role in the antioxidant activity of protein hydrolysates; thus, it is necessary to select an appropriate protease [10]. In this section, six proteases were used to hydrolyze oyster meat, and the optimum protease hydrolysates were expected to show higher hydroxyl-radical-scavenging activity and DH and BCAA contents. As shown in Figure 1, the data were evaluated by analysis of variance (ANOVA) using SPSS software. The oyster meat hydrolysate treated with trypsin demonstrated the highest DH content of 10.54% and BCAA content of 17.93%, values that were significantly higher than the other five protease hydrolysates (*p* < 0.05). Furthermore, different enzyme hydrolysates exhibited different antioxidant activities because of the restriction enzyme sites were different. The order of hydroxyl-radical-scavenging activity for the six hydrolysates was trypsin > protamex > neutrase > flavourzyme > bromelain > papain. This was consistent with the result that the hydrolysates with relatively high DHs showed stronger antioxidant activity than those with low DHs [14], and some studies have shown that BCAA content is related to oxidative stress in mice [15]. In summary, the trypsin hydrolysate showed the highest biological activity of the six protease hydrolysates, and trypsin was chosen as the best candidate for further study.

Furthermore, the optimum enzyme conditions may also depend on the response values; for example, neutrase has an optimum temperature of 39.55 °C when the hydrolysate has a higher DPPH-scavenging activity, and an optimum temperature of 43.72 °C when the hydrolysate has higher cellular antioxidant activity [16,17]. The hydroxyl-radical-scavenging activity and DH and BCAA contents are related, and hydrolysates with different response values may be applied to different research fields. Based on the above background, we optimized the preparation conditions of the OPs obtained with different response values and obtain the target OP with high activity.

### 2.2. Single-Factor Experiments

Enzymatic hydrolysis is influenced by several factors, such as enzyme concentration, pH, time, temperature, and water/material ratio, which influence the degree of enzymatic hydrolysis reaction [18]. As shown in Table 1, the optimal conditions of five single factors on the hydroxyl-radical-scavenging activity and DH and BCAA contents of the OP were summarized. The optimal conditions for Oyster Peptide 1 (OP-1) were an enzyme concentration of 1500 U/g, a pH of 8.0, a hydrolysis temperature of 40 °C, a hydrolysis time of 6.0 h, and a water/material ratio of 8.0; Oyster Peptides 2 (OP-2) and 3 (OP-3) had higher hydrolysis temperatures and lower water/material ratios than OP-1.

### 2.3. Optimization of Hydrolysis Conditions by BBD

RSM was employed to investigate the effects of the independent variables on oyster hydrolysates. Based on the single-factor experiments, the five independent variables used in BBD by RSM were listed in Table 2. The results showed that the DH content of the OP ranged from 5.91% to 10.45%, and the polynomial equation was as follows:

DH(%) = +10.13 + 0.38A + 0.20B + 0.054C + 0.037D + 0.28E + 0.21AB − 0.074AC + 0.19AD − 0.005AE + 0.23BC − 0.12BD + 0.47BE − 0.036CD − 0.065CE − 0.18DE − 1.09A^2^ − 0.96B^2^ − 1.77C^2^ − 1.28D^2^ − 1.93E^2^ (1)

Table 2 also shows that the hydroxyl-radical-scavenging activity of the OP ranged from 36.76% to 70.85% and the BCAA content of the OP ranged from 16.73% to 18.15%. The polynomial equations by BBD for hydroxyl-radical-scavenging activity and BCAA content of OP were as follows:

Hydroxyl-radical-scavenging activity (%) = +64.90 + 0.22A + 6.81B + 0.59C − 2.49D − 2.87E − 1.19AB + 1.50AC − 3.34AD − 6.09AE − 5.55BC + 4.91BD + 0.27BE − 0.43CD − 0.46CE − 4.30DE − 3.15A^2^ − 5.18B^2^ − 0.42C^2^ − 8.16D^2^ − 9.63E^2^ (2)

BCAA(%) = +17.44 + 0.086A + 0.023B − 0.21C + 0.18D − 0.16E − 0.12AB + 0.16AC − 0.065AD + 0.39AE − 0.077BC + 0.021BD + 0.092BE − 0.19CD + 0.26CE + 0.13DE − 0.15A^2^ + 0.039B^2^ − 0.12C^2^ + 0.11D^2^ + 0.15E^2^ (3)

The analysis of variance (ANOVA) results for the model are given in Table 3. The model F-value implies that the models were significant, and Prob > *F* values of less than 0.0500 indicate that the model terms were significant [17]. As shown in Table 3, the *p*-values for the three models of DH content, hydroxyl-radical-scavenging activity, and BCAA content were all <0.01, which indicated that all the models were significant; the lack of fit values of the three models were 0.6612, 0.4923, and 0.4359, which further indicated that the models were significant. Furthermore, in the DH content model, independent variables A, B, E, BE, A^2^, B^2^, C^2^, D^2^, and E^2^ were significant; in the hydroxyl-radical-scavenging activity model, independent variables B, D, E, AD, AE, BC, BD, DE, A^2^, B^2^, D^2^, and E^2^ were significant; and in the BCAA content model, independent variables A, C, D, E, AB, AC, AE, BC, BE, CD, CE, DE, A^2^, C^2^, D^2^, and E^2^ were significant.

R^2^ was defined as the ratio of the explained variation to the total variation and was a measurement of the degree of fitness [12]. The R^2^ values for the DH content, hydroxyl-radical-scavenging activity, and BCAA content models were 0.9732, 0.9523, and 0.9749, respectively, and the Pred R^2^ values were in reasonable agreement with the Adj R^2^ values. The findings suggested that the DH content, hydroxyl-radical-scavenging activity and BCAA content could be analyzed and predicted by the model.

Determined using Design-Expert 8.0, the optimal hydrolysis conditions of the OP with different response values are shown in Table 4. The highest DH of OP (9.85 ± 0.76)% was obtained at an enzyme concentration of 1593.2 U/g, a pH of 8.2, a hydrolysis temperature of 40.1 °C, a hydrolysis time of 6.0 h, and a water/material ratio of 8.2. The highest hydroxyl-radical-scavenging activity of OP (70.12 ± 2.37)% was obtained at an enzyme concentration of 1546.3 U/g, a pH of 9.0, a hydrolysis temperature of 50.2 °C, a hydrolysis time of 5.1 h, and a water/material ratio of 5.6. Finally, the highest BCAA content of OP (17.88 ± 1.28)% was obtained at an enzyme concentration of 1323.8 U/g, a pH of 8.3, a hydrolysis temperature of 41.7 °C, a hydrolysis time of 6.7 h, and a water/material ratio of 4.8. The three experimental values were in agreement with the predicted values of 10.20%, 70.91%, and 18.15%, respectively.

### 2.4. Separation and Purification of the OP

**Figure 2:**
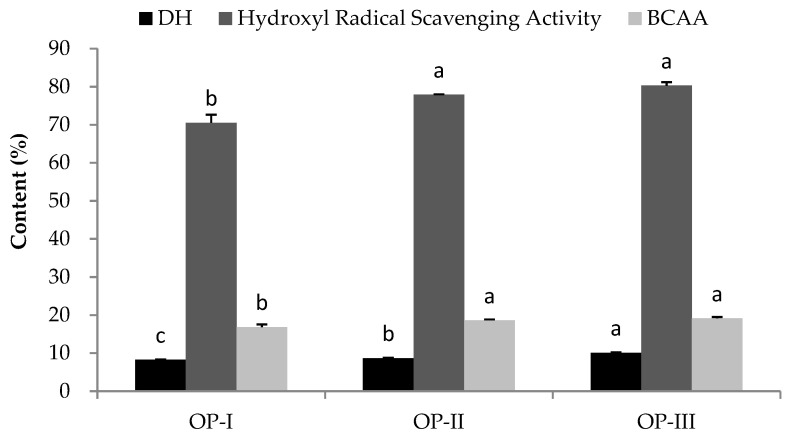
The content of the parameters of different fractions from the OP. Different letters indicate significant differences between groups (*p* < 0.05).

The OP was separated into three fractions by the membranes with MWCOs of 10,000 Da and 3500 Da: OP-I (>10,000 Da), OP-II (3500–10,000 Da), and OP-III (<3500 Da). As shown in Figure 2, OP-III exhibited the highest DH with a value of (10.10 ± 0.12)%, which was significantly higher than those of OP-I and OP-II (*p* < 0.05). The values of hydroxyl-radical-scavenging activity and BCAA content of OP-III were (80.28 ± 0.92)% and (19.14 ± 0.36)%, respectively, which were higher than those of the other two fractions. Therefore, OP-III was used for further antioxidant assays and purification.

**Figure 3:**
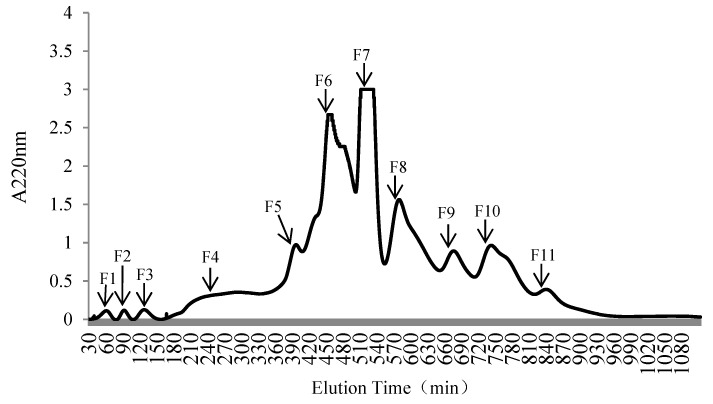
Separation chromatogram for OP-III, using a Sephadex G-25 gel filtration column.

**Figure 4:**
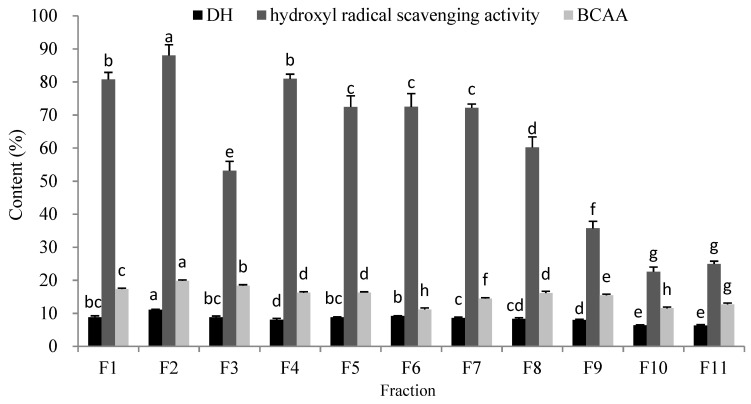
The contents of the parameters of different fractions from OP-III. Different letters indicate significant differences between groups (*p* < 0.05).

OP-III was further fractioned with a Sephadex G-25 gel filtration column. The elution time was 18 h, and 11 fractions, designated F1–F11, were obtained (Figure 3). The 11 fractions were collected and freeze-dried to assess DH, hydroxyl-radical-scavenging activity, and BCAA analysis. Figure 4 showed that Fraction F2 exhibited the highest DH, hydroxyl-radical-scavenging activity, and BCAA content, with values of (11.08 ± 0.11)%, (88.04 ± 3.23)%, and (19.85 ± 0.25)%, respectively, which were significantly higher than those of the other ten fractions (*p* < 0.05). Finally, the purified peptide F2 was obtained from the oyster protein hydrolysate, and its amino acid sequence was determined by LC-MS/MS.

As shown in Table 5, 13 high-scoring peptides were identified. The results showed that the oyster protein could be hydrolyzed into high-molecular-weight peptides and not only short peptides. The masses of the peptides ranged from 831.43 Da to 1557.74 Da, and their sequences were between 7 and 13 amino acids in length.

## 3. Discussion

Based on the above data, different response values were obtained for hydrolysates generated with different independent variable conditions. In our DH assay study, the DH content of the OP increased significantly when the hydrolysis time increased from 5.0 h to 6.0 h and decreased significantly with a hydrolysis time from 6.0 h to 7.0 h. This trend was consistent with the results of Wang et al. [12], which showed that the DH of fish gelatin hydrolysate increased significantly (*p* < 0.05) when the time increased from 1.0 h to 2.0 h, and decreased slowly when the time increased from 2.0 h to 3.0 h. We inferred that the hydrolysis reaction was powerful in the first few hours because of the sufficient raw material and enzyme quantity, and the reaction flattened later with the consumption of raw material and protease [19]. Some studies have come to different conclusions; for example, Zheng et al. [20] found that the DH value obtained from alcalase increased rapidly during the first 2.0 h of the reaction and then slowly increased, while the hydrolysates obtained from bromelain achieved an obvious increase after 2.0 h. Tan et al. [21] found that the DH of cod bone protein increased rapidly during the first 0.5 h because more peptide bonds were broken, and then the DH showed a slow increase. The DH may be different in various studies because of the different marine organism parts and different enzymes that were used in hydrolysis [22]. In the BCAA content assay, the BCAA content of the OP increased from 17.40% to 18.40% when the hydrolysis time increased from 5.0 h to 7.0 h, and the hydroxyl-radical-scavenging activity of the OP increased when the hydrolysis time increased from 4.0 h to 5.0 h before leveling off, which was consistent with the report from Wang et al. [12], who found that the DPPH-radical-scavenging activity of fish gelatin hydrolysate increased significantly (*p* < 0.05) when the hydrolysis time increased from 1.0 h to 2.0 h, and then increased slightly when the hydrolysis time increased from 2.0 h to 3.0 h. In our previous study, we found that the DPPH-scavenging activity of mackerel protein hydrolysate increased slightly when the hydrolysis time increased from 4.0 h to 5.0 h and then decreased slightly with a hydrolysis time of 6.0 h [16].

Hydrolysis temperature was another important independent variable. We found that the DH content of OP increased significantly and then decreased significantly when the hydrolysate temperature increased from 35 °C to 45 °C, while the hydroxyl-radical-scavenging activity of the OP decreased with temperature from 40 °C to 60 °C. We speculated that the enzymes were irreversibly denatured at high temperatures [23]. Moreover, at higher temperatures, the increased heat-inactivation rate led to a faster decrease in the number of active catalyst molecules [24]. The result was different from that reported by Ji et al. [25], who found a linear parallel relationship between the DH and the antioxidant properties. In their study, the DH content increased rapidly with an increase in temperature from 30 °C to 60 °C, because different enzymes had different optimal hydrolysate temperatures. Additionally, the change of BCAAs in the OP content had the same tendency of change as the hydroxyl-radical-scavenging activity. When the hydrolysis time was 7.0 h and the enzyme concentration was 1000 U/g, the BCAA content of OP decreased sharply when the temperature increased from 40 °C to 60 °C.

Furthermore, in the DH assay, the DH content of the OP first increased and then decreased as the enzyme concentration, pH, and water/material ratio increased. Excess enzyme might not participate in the reaction; if so, once a certain enzyme concentration is reached, the DH content would plateau. The hydroxyl-radical-scavenging activity of OP increased slightly, and the BCAA content of OP decreased with increasing enzyme concentration, which could be explained by the fact that the higher enzyme concentration saturated the hydrolysates [24]. A sharp increase in the hydroxyl-radical-scavenging activity and BCAA content of OP was achieved by increasing the pH, especially the initial pH, which showed a significant effect on the response. The report by Guerard et al. [26] also showed a similar effect of pH on the hydrolysis of shrimp-processing discards.

When the water/material ratio increased, the hydroxyl-radical-scavenging activity of OP first increased when the water/material ratio increased from 4.0 to 7.0, and then decreased when the water/material ratio further decreased from 7.0 to 8.0. This result was consistent with that reported by Feng et al. [27]. We speculated that a higher water/material ratio might dilute the enzyme concentration and slow the rate of the enzymatic reaction, while the BCAA content of OP decreased slightly when the water/material ratio increased.

Furthermore, OP-III, with a molecular weight below 3500 Da, exhibited high hydroxyl-radical-scavenging activity, which was consistent with previous studies that confirmed that peptides below 3000 Da exhibit higher antioxidant activity. Ann et al. [28] found that salmon-byproduct peptides in the range of 1000 Da–2000 Da exhibited the highest antioxidant activity; Dong et al. [29] prepared peptides from oyster meat, and fractions below 1000 Da showed the strongest antioxidant activity; these results showed that the antioxidant activity of peptides is dependent on their molecular weight. In contrast, Hamzeh et al. [30] prepared a cuttlefish (*Sepia pharaonis*) peptide and found that the peptide with a molecular weight of 3000 Da–10,000 Da had the greatest DPPH radical scavenging activity, the 10,000 Da–30,000 Da fraction had the highest reducing power, and the <3000 Da fraction had the greatest ABTS-radical-scavenging activity. Therefore, we should conduct more OP antioxidant activity assay in future studies.

Furthermore, the DH and BCAA contents were higher in the target fraction when the hydroxyl-radical-scavenging activity was higher in our study. Some studies have confirmed that hydrolysates with high DH contents exhibit greater antioxidant activity [31], and the crude protein and amino acid contents increased with increasing DH values [32]. Finally, we obtained 13 peptides from OP-III. The purified peptides no. 1 to no. 6 were rich in Lys residues, and the C-termini of these peptides contained Lys residues. Some studies have confirmed that Lys may contribute to the radical-scavenging activity of purified peptides [33,34]. It was also reported that Arg may play an important role in the antioxidative activity of peptides [35]. Furthermore, previous studies have reported that peptides with amino acids such as His and Thr had high antioxidant activity [36,37]. Therefore, in this study, the purified peptides consisting of the above amino acid residues may have contributed to their higher radical-scavenging potential. It should be noted that the OP-III-F2 was also a cocktail of water-soluble solids and contained some small-size glycogen and minerals, which would affect its antioxidant activity. Further purification work is needed to obtain the single purified peptide.

## 4. Material and Methods

### 4.1. Materials and Chemicals

Oyster (*C. talienwhanensis*) was purchased from a seafood market in Qingdao, China. Upon arrival, the oyster was washed, and the edible meat was separated from the shells, homogenized, and stored at −20 °C until use.

Six proteases (compound proteinase, trypsin, bromelain, papain, neutrase, and flavourzyme) were purchased from Beijing Solarbio Science & Technology Co., Ltd. (Beijing, China). The ultrafiltration (UF) system and UF membranes with molecular weight cut-offs (MWCO) of 10,000 Da and 3500 Da were purchased from Laungy Co., Ltd. (Shanghai, China). The fraction collector and computer ultraviolet (UV) detector were purchased from Shanghaihuxi Analysis Instrument Factory Co., Ltd. (Shanghai, China). The water was distilled and purified using a Milli-Q Water Purification System (Millipore, Bedford, MA, USA). All other chemicals and solvents were of analytical grade.

### 4.2. Preparation of Oyster Protein Hydrolysates

Minced oyster was mixed with deionized water at a ratio of 1:10 *v*/*w*. The mixtures were adjusted to the required pH and heated in a water bath to the required temperature before the six proteases (protamex, trypsin, bromelain, papain, neutrase, and flavourzyme) were added in proper proportions based on enzymatic activity. The hydrolysis reactions were performed in a shaking incubator. At the end of the hydrolysis period, the mixtures were heated in boiling water for 10 min to inactivate the proteases. Next, the hydrolysates were centrifuged at 18,000 × g (4 °C) for 30 min and the supernatants were stored at 4 °C before use.

### 4.3. Single-Factor Experiments

In this section, the trypsin-treated hydrolysate was chosen as the best candidate, and five independent variables (enzyme concentration, pH, hydrolysis temperature, hydrolysis time, water/material ratio) were selected for the single-factor experiments. The hydrolysis condition of trypsin was an enzyme concentration of 1000 U/g, a pH of 8.0, a hydrolysis temperature of 40 °C, a hydrolysis time of 6.0 h, and a water/material ratio of 10. The ranges of tested variables were as follows: enzyme concentrations of 500, 1000, 1500, 2000, 2500, and 3000 U/g; pH of 7.0, 7.5, 8.0, 8.5, and 9.0; hydrolysis temperatures of 35, 40, 45, 50, 55, and 60 °C; hydrolysis times of 3.0, 4.0, 5.0, 6.0, 7.0, and 8.0 h; and water/material ratios of 2, 4, 6, 8, and 10. While searching for the optimal condition of one variable, the values of other variables were fixed. The method of enzymatic hydrolysis is described in Section 4.2.

### 4.4. Optimization of Oyster Protein Hydrolysate Preparative Conditions

Extraction optimization in general was performed using RSM. On the basis of the single-factor experiments, the five independent variables at five levels were designed in a Box-Behnken design (BBD) by RSM. Design Expert (trial version 8.0.6; State-Ease Inc., Minneapolis, MN, USA) was used to analyze and calculate the predicted responses and experimental design for the cellular antioxidant activity. The responses obtained from each set of experimental designs were analyzed via multiple regressions to fit the following quadratic polynomial model.

(4) $$Y=\beta_{0}+\sum_{i=1}^{k}\beta_{i}X_{i}+\sum_{i=1}^{k}\beta_{ii}X_{i}^{2}+\sum \sum_{i<j}\beta_{ij}X_{i}X_{j}$$

where *Y* is the response variable; $\beta_{0}$ is a constant; $\beta_{i}$, $\beta_{ii}$, and $\beta_{ij}$ are the linear, quadratic, and interaction coefficients, respectively; and $X_{i}$ and $X_{j}$ are the coded independent variables.

According to Design Expert software, an analysis of variance table was generated, and the effect and regression coefficients of the linear, quadratic and interaction terms were determined. *p*-Values greater than 0.05 indicated that the model terms were not significant. The regression coefficient was used to perform statistical calculations, and the generated 3D surface was determined from the fitted polynomial equation.

### 4.5. Degree of Hydrolysis

The degree of hydrolysis (DH) was evaluated as the proportion (%) of amino nitrogen with respect to the total nitrogen in the sample [38]. Analyses were performed in duplicate.

(5) $$\mathrm{DH}\left(\%\right)=\frac{10\;\%\text{TCA soluble nitrogen in the sample}}{\text{total nitrogen in the sample}}\times 100\%$$

### 4.6. Hydroxyl-Radical-Scavenging Activity

The hydroxyl-radical-scavenging activity of the hydrolysates was tested using the method described by You et al. [39], with several modifications. Briefly, the reaction mixture contained 1.0 mL of phosphate buffer (PBS, 0.15 mol/L, pH 7.4), 1.0 mL of safranine T (1.0 mM), 0.5 mL of EDTA-FeSO_4_ (2.0 mmol/L), and 1.0 mL of hydrolysates at a certain concentration. After sufficient mixing, 1.0 mL of H_2_O_2_ was added to the mixture. Following incubation at 37 °C for 30 min, the absorbance of the mixture was measured at 520 nm. The hydroxyl-radical-scavenging activity was calculated as scavenging rate (%) = ((A_1_ − A_0_)/(A_2_ − A_0_)) × 100, where A_1_ was the absorbance of the hydrolysates with H_2_O_2_, A_2_ was the absorbance without H_2_O_2_, and A_0_ was the absorbance of the control. Both A_0_ and A_2_ were mixtures with the sample solution replaced by deionized water. All experiments were performed in triplicate.

### 4.7. Amino Acid Composition Analysis

Amino acid content was measured after acid hydrolysis in accordance with Luo et al. [40], with some modifications. Hydrolysis of the sample was conducted with 6 M HCl at 110 °C for 24 h in a drying oven; the sample was then transferred into a 50 mL volumetric flask and diluted to the reticule with distilled water. Next, 1 mL of the filtrate was evaporated at 40–50 °C with a rotary evaporator, dissolved in 1–2 mL of distilled water, and dried. After complete drying, the residue was dissolved in 1 mL of buffer (pH 2.2) and subjected to analysis on an S433D amino acid analyzer (SYKAM, Eresing, Germany). Amino acids were identified and quantified from constructed standard curves.

BCAAs include the amino acids valine (Val), leucine (Leu), and isoleucine (Ile). The BCAA content of the hydrolysates was calculated as BCAA (%) = (the sum of Val, Leu, and Ile content)/total amino acids ×100.

### 4.8. Purification of Oyster Protein Hydrolysates

#### 4.8.1. Ultrafiltration

The oyster protein hydrolysate was fractionated through UF membranes with MWCOs of 10,000 Da and 3500 Da using an Amicon model LNG-UF-101, and the three fractions were freeze-dried for further studies.

#### 4.8.2. Gel Filtration Chromatography

The fraction described above was further purified using a gel filtration column (SephadexG-25 fine, 2.6 × 80 cm, GE Healthcare Bio-Science AB, Uppsala, Sweden). The oyster peptide (OP) (0.5 g) was dissolved in distilled water (2 mL), and the mixture was filtered through a 0.22 µm filter membrane (25 mm id.). The mobile phase of the column was Milli-Q water, and the flow rate was 0.5 mL/min. The eluted fraction was collected and monitored at 220 nm using a computerized ultraviolet detector (model HD-5T). The fractions were freeze-dried for further studies.

#### 4.8.3. Identification of OP by Mass Spectrometry

The final peptide compositions were identified by LC-MS/MS according to the method of Wang et al. [17], with some modifications. The target peptide was further separated on an Acclaim PepMap RSLC column (C18, 5 μm, 100 Å, 300 μm × 5 mm) with a 60 min gradient of 5 min 6–9% Buffer B (80% ACN, 0.1% formic acid), 15 min 9–14% Buffer B, 30 min 14–30% Buffer B, 8 min 30–40% Buffer B, and 2 min 40–95% Buffer B. Peptides were analyzed using a Thermo Scientific Q Exactive mass spectrometer in data-dependent mode, with an automatic switch between MS and MS/MS scans using a top 20 method. Instrument parameters were as follows: resolution 70,000 for full MS scan and 75,000 for MS2 scan, automatic gain control target 3e6 for full scan and 1e5 for MS2, and maximum ion injection time of 40 ms for full MS scan and 60 ms for MS2 scan.

### 4.9. Statistical Analysis

Data are presented as the means ± SD. Statistical significance of the data was determined by ANOVA using SPSS software (version 18.0 for Windows, SPSS Inc., Chicago, IL, USA) and means were compared by Duncan’s multiple comparison post-test. Differences were considered to be significant at *p* < 0.05.

## 5. Conclusions

To utilize the oyster (*C. talienwhanensis*) protein sufficiently, the OP prepared by trypsin exhibited the highest DH of (9.85 ± 0.76)%, the highest hydroxyl-radical-scavenging activity of (70.12 ± 2.37)%, and the highest BCAA content of (17.88 ± 1.28)%. Statistical analyses based on BBD by RSM showed that the three experimental values of the OP were in agreement with the predicted values of 10.20%, 70.91%, and 18.15%, respectively, suggesting a good fit between the models and the experimental data. Three different OP preparation conditions provided the theoretical basis for the high-value utilization of oysters. Furthermore, we used oyster peptide to purify the OP, and 13 amino acid sequences were identified from OP-III by LC-MS/MS. Further studies will be performed to purify and identify more peptides from the OP, and more detailed studies on the bioactivities and structure–activity relationships of the purified peptides will also be needed.

## Figures and Tables

**Table 1:** The optimum conditions for single-factor experiments on the DH, hydroxyl radical scavenging activity, and BCAA.

Numbers	Response Values	A (U/g)	B	C (°C)	D (h)	E (*w*/*v*)
OP-1	DH	1500	8.0	40	6.0	8.0
OP-2	Hydroxyl radical Scavenging activity	1500	8.5	55	5.0	6.0
OP-3	BCAA	1500	8.0	50	6.0	6.0

A, B, C, D, and E indicate the enzyme concentration (U/g), pH, hydrolysis temperature (°C), hydrolysis time (h), and water/material ratio (*w*/*v*), respectively.

**Table 2:** Experimental design and result of different response values.

Numbers	A (U/g)	B		C (°C)			D (h)		E (*w*/*v*)		Y1: DH (%)	Y2: Hydroxyl-Radical-Scavenging Activity (%)	Y3: BCAA (%)
		Y1/Y3	Y2	Y1	Y2	Y3	Y1/Y3	Y2	Y1	Y2/Y3			
1	1000	7	8	40	55	50	6	5	8	6	7.71	45.90	17.08
2	2000	7	8	40	55	50	6	5	8	6	8.05	49.21	17.55
3	1000	9	9	40	55	50	6	5	8	6	7.66	63.79	17.36
4	2000	9	9	40	55	50	6	5	8	6	8.85	62.33	17.35
5	1500	8	8.5	35	50	40	5	4	8	6	7.12	55.92	17.25
6	1500	8	8.5	45	60	60	5	4	8	6	7.08	58.91	17.19
7	1500	8	8.5	35	50	40	7	6	8	6	7.23	49.82	18.00
8	1500	8	8.5	45	60	60	7	6	8	6	7.05	55.10	17.16
9	1500	7	8	40	55	50	6	5	6	4	7.44	47.56	17.84
10	1500	9	9	40	55	50	6	5	6	4	6.95	57.08	17.72
11	1500	7	8	40	55	50	6	5	10	8	7.12	44.57	16.94
12	1500	9	9	40	55	50	6	5	10	8	8.51	54.40	17.62
13	1000	8	8.5	35	50	40	6	5	8	6	6.92	63.76	17.41
14	2000	8	8.5	35	50	40	6	5	8	6	7.45	62.22	17.35
15	1000	8	8.5	45	60	60	6	5	8	6	7.38	60.55	16.73
16	2000	8	8.5	45	60	60	6	5	8	6	7.62	64.99	17.30
17	1500	8	8.5	40	55	50	5	4	6	4	6.39	51.30	17.88
18	1500	8	8.5	40	55	50	7	6	6	4	6.93	52.97	17.95
19	1500	8	8.5	40	55	50	5	4	10	8	7.24	52.27	17.28
20	1500	8	8.5	40	55	50	7	6	10	8	7.05	36.76	17.87
21	1500	7	8	35	50	40	6	5	8	6	7.47	46.76	17.49
22	1500	9	9	35	50	40	6	5	8	6	7.20	70.85	17.65
23	1500	7	8	45	60	60	6	5	8	6	7.01	60.68	17.23
24	1500	9	9	45	60	60	6	5	8	6	7.65	62.57	17.08
25	1000	8	8.5	40	55	50	5	4	8	6	7.51	47.16	17.16
26	2000	8	8.5	40	55	50	5	4	8	6	8.20	56.98	17.34
27	1000	8	8.5	40	55	50	7	6	8	6	7.25	53.76	17.58
28	2000	8	8.5	40	55	50	7	6	8	6	8.69	45.21	17.50
29	1500	8	8.5	35	50	40	6	5	6	4	5.91	52.22	18.15
30	1500	8	8.5	45	60	60	6	5	6	4	6.27	53.13	17.17
31	1500	8	8.5	35	50	40	6	5	10	8	6.65	48.95	17.21
32	1500	8	8.5	45	60	60	6	5	10	8	6.75	49.81	17.28
33	1000	8	8.5	40	55	50	6	5	6	4	6.25	49.28	17.84
34	2000	8	8.5	40	55	50	6	5	6	4	7.06	62.71	17.23
35	1000	8	8.5	40	55	50	6	5	10	8	6.75	54.25	16.80
36	2000	8	8.5	40	55	50	6	5	10	8	7.54	40.93	17.73
37	1500	7	8	40	55	50	5	4	8	6	7.34	49.71	17.33
38	1500	9	9	40	55	50	5	4	8	6	8.15	51.79	17.36
39	1500	7	8	40	55	50	7	6	8	6	7.54	36.92	17.71
40	1500	9	9	40	55	50	7	6	8	6	7.88	60.65	17.83
41	1500	8	8.5	40	55	50	6	5	8	6	10.45	62.45	17.35
42	1500	8	8.5	40	55	50	6	5	8	6	10.34	60.60	17.49
43	1500	8	8.5	40	55	50	6	5	8	6	9.86	63.82	17.20
44	1500	8	8.5	40	55	50	6	5	8	6	9.93	66.14	17.23
45	1500	8	8.5	40	55	50	6	5	8	6	10.33	58.39	17.40
46	1500	8	8.5	40	55	50	6	5	8	6	9.89	65.88	17.49

A, B, C, D, and E indicate the enzyme concentration (U/g), pH, hydrolysis temperature (℃), hydrolysis time (h), and water/material ratio (*w*/*v*), respectively. Y1 indicates the DH content, Y2 indicates hydroxyl-radical-scavenging activity, and Y3 indicates the BCAA content.

**Table 3:** ANOVA for response surface quadratic model.

Response Values	Model	*p*-Value	Lack of Fit	Predicted R^2^	Adj R^2^	R^2^
	*F*-Value	Prob > F	*p*-Value			
DH (%)	45.31	<0.0001	0.6612	0.9086	0.9517	0.9732
Hydroxyl-radical-scavenging activity (%)	24.96	<0.0001	0.4923	0.8314	0.9142	0.9523
BCAA (%)	48.61	<0.0001	0.4359	0.9104	0.9549	0.9749

**Table 4:** Optimal conditions of hydrolysis and model validation.

Response Values	A (U/g)	B	C (℃)	D (h)	E (*w*/*v*)	Predicted Value	Experimental Value
DH	1593.2	8.2	40.1	6.0	8.2	10.20	9.85 ± 0.76
Hydroxyl-radical-scavenging activity	1546.3	9.0	50.2	5.1	5.6	70.91	70.12 ± 2.37
BCAA	1323.8	8.3	41.7	6.7	4.8	18.15	17.88 ± 1.28

A, B, C, D, and E indicate the enzyme concentration (U/g), pH, hydrolysis temperature (°C), hydrolysis time (h), and water/material ratio (*w*/*v*), respectively.

**Table 5:** The peptide sequences of F2.

No.	Sequence	Mass (Da)	Length	Parental Protein	Position
1	LAGELHQEQENYK	1557.74	13	K1QTC1	745–757
2	AIDTIINQK	1014.57	9	K1R6Z7	223–231
3	DSYVGDEAQSK	1197.52	11	Q8TA69;C4NY62;	52–62
4	PGTTEDEPVK	1071.51	10	K1Q5P0	498–507
5	ETVIDTIQK	1045.57	9	K1RHA0	148–156
6	DLESQLK	831.43	7	K1QRU8	989–995
7	NAETELGETSQR	1333.61	12	K1QTC1	681–692
8	EYDESGPSIVHR	1387.64	12	Q8TA69;C4NY62	362–373
9	DSDLEGHPTPR	1222.56	11	K1RBC9	173–183
10	HDNPGDLGDLH	1188.52	11	K1PY89;K1QLW5	128–138
11	AQCEMEPNH	1114.42	9	K1PY89;K1QLW5	47–55
12	ESAGIHETT	943.42	9	Q8TA69	271–279
13	NTVLSGGTT	848.42	9	Q8TA69	297–305
